# Azathioprine Biotransformation in Young Patients with Inflammatory Bowel Disease: Contribution of Glutathione-S Transferase M1 and A1 Variants

**DOI:** 10.3390/genes10040277

**Published:** 2019-04-04

**Authors:** Marianna Lucafò, Gabriele Stocco, Stefano Martelossi, Diego Favretto, Raffaella Franca, Noelia Malusà, Angela Lora, Matteo Bramuzzo, Samuele Naviglio, Erika Cecchin, Giuseppe Toffoli, Alessandro Ventura, Giuliana Decorti

**Affiliations:** 1 Centro di Riferimento Oncologico, IRCCS, 33081 Aviano, Italy; mlucafo@units.it (M.L.); ececchin@cro.it (E.C.); gtoffoli@cro.it (G.T.); 2 Institute for Maternal and Child Health IRCCS Burlo Garofolo, 34137 Trieste, Italy; stefano.martelossi@aulss2.veneto.it (S.M.); dieg.o@libero.it (D.F.); angelalora86@gmail.com (A.L.); matteo.bramuzzo@burlo.trieste.it (M.B.); samuele.naviglio@burlo.trieste.it (S.N.); alessandro.ventura@burlo.trieste.it (A.V.); decorti@units.it (G.D.); 3 Department of Life Sciences, University of Trieste, 34127 Trieste, Italy; 4 Department of Medical, Surgical and Health Sciences, University of Trieste, 34149 Trieste, Italy; rfranca@units.it; 5 Sanitary Services Agency 1, 34129 Trieste, Italy; tossicologia.forense@asuits.sanita.fvg.it

**Keywords:** azathioprine, inflammatory bowel disease, glutathione-S transferase, pharmacogenetics, pharmacokinetics

## Abstract

The contribution of candidate genetic variants involved in azathioprine biotransformation on azathioprine efficacy and pharmacokinetics in 111 young patients with inflammatory bowel disease was evaluated. Azathioprine doses, metabolites thioguanine-nucleotides (TGN) and methylmercaptopurine-nucleotides (MMPN) and clinical effects were assessed after at least 3 months of therapy. Clinical efficacy was defined as disease activity score below 10. Candidate genetic variants (*TPMT* rs1142345, rs1800460, rs1800462, *GSTA1* rs3957357, *GSTM1*, and *GSTT1* deletion) were determined by polymerase chain reaction (PCR) assays and pyrosequencing. Statistical analysis was performed using linear mixed effects models for the association between the candidate variants and the pharmacological variables (azathioprine doses and metabolites). Azathioprine metabolites were measured in 257 samples (median 2 per patient, inter-quartile range IQR 1-3). Clinical efficacy at the first evaluation available resulted better in ulcerative colitis than in Crohn’s disease patients (88.0% versus 52.5% responders, *p* = 0.0003, linear mixed effect model, LME). TGN concentration and the ratio TGN/dose at the first evaluation were significantly higher in responder. *TPMT* rs1142345 variant (4.8% of patients) was associated with increased TGN (LME *p* = 0.0042), TGN/dose ratio (LME *p* < 0.0001), decreased azathioprine dose (LME *p* = 0.0087), and MMPN (LME *p* = 0.0011). *GSTM1* deletion (58.1% of patients) was associated with a 18.5% decrease in TGN/dose ratio (LME *p* = 0.041) and 30% decrease in clinical efficacy (LME *p* = 0.0031). *GSTA1* variant (12.8% of patients) showed a trend (*p* = 0.046, LME) for an association with decreased clinical efficacy; however, no significant effect on azathioprine pharmacokinetics could be detected. In conclusion, GSTs variants are associated with azathioprine efficacy and pharmacokinetics.

## 1. Introduction

Inflammatory bowel disease (IBD) is a chronic, relapsing and remitting disease of the gastrointestinal tract that comprises two main entities, Crohn’s disease (CD) and ulcerative colitis (UC). The disease has a peak onset in subjects 15 to 30 years old, and its incidence is rising in the pediatric population [1]. Despite the recent introduction in therapy of biologicals, thiopurines continue to be widely used in this disease; indeed, these are cheap drugs, and maintain at least 20% of patients in a state of stable long term steroid free clinical remission [2]. Among thiopurines, azathioprine is mainly used as an immunosuppressant in IBD, and, although it has a well described risk benefit profile, adverse drug reactions are relatively common, occurring in 15–18% of patients, and can be severe enough to require the withdrawal of therapy [3,4] In addition, a significant proportion of patients does not respond to therapy with this agent [5,6,7]. The reasons of this high heterogeneity in clinical response is not clear yet [8]; however, variability in azathioprine metabolism can be important; indeed, azathioprine is a prodrug that requires metabolic conversion to its active form. The first step in this metabolic conversion is mediated by conjugation with glutathione, resulting in the formation of mercaptopurine. This reaction is in part nonenzymatic but it is even controlled, as demonstrated by recent publications, by the enzyme glutathione S-transferase (GST in particular by the isoforms A and M [9]. The latter is also inactive and is converted by the enzymes of the purine salvage pathway to the active thioguanine nucleotides, which are responsible of the cytotoxic and apoptotic effect of these drugs [10]. Mercaptopurine is metabolized by the enzyme xanthine oxidase in the liver, and by thiopurine methyltransferase (TPMT) and inosine triphosphatase (ITPA), mainly in extra hepatic tissues [10]. Polymorphisms in genes involved in azathioprine metabolism can hence influence the efficacy and toxicity of this drug [6].

In this study, we aimed to evaluate the contribution of candidate genetic variants involved in azathioprine biotransformation on azathioprine efficacy and pharmacokinetics in young patients with IBD.

## 2. Materials and Methods

### 2.1. Patient Characteristics

111 patients with IBD were enrolled by the Gastroenterology Unit of the Pediatric Hospital “Burlo Garofolo” in Trieste, Italy between March 2004 and February 2015. The study was conducted in accordance with the Declaration of Helsinki, and the protocol was approved by the Institutional Ethics Committee (Projects RC 23/2005 and 12/2013). All subjects and parents gave their informed consent for inclusion before they participated in the study. The inclusion criteria were age less than 30 years, previous diagnosis of IBD and treatment with azathioprine for at least 3 months. The patients enrolled are all the patients taking azathioprine at “Burlo Garofolo” in Trieste in the time-frame of the study. Blood samples for azathioprine metabolites measurement and for genotyping were taken at the appropriate clinic visit. Timing of metabolite level measurement was determined by the clinical setting of azathioprine administration at the hospital: generally, azathioprine metabolites levels were measured after 3, 6, and 12 months of treatment and then every year. Patients were treated with a dose-escalating strategy to reduce the risk of adverse events starting, however, from a relatively high dose (median of 2 mg/kg). At subsequent follow-up visits (every 3 months), the dose was increased or reduced so as to obtain the optimal clinical response; the criteria used to increase or reduce the dose of azathioprine were the level of disease activity and laboratory parameters used to monitor azathioprine toxicity (in particular leukocyte, erythrocyte and platelet counts, hemoglobin concentration, mean corpuscular volume, liver enzymes alanine aminotransferase, aspartate aminotransferase and γ-glutamyltransferase, and amylase levels). According to current guidelines, genotyping information was shared with the clinicians only for patients presenting *TPMT* variant alleles, in order to allow increased monitoring of adverse events. 

Clinical response was assessed using Pediatric Crohn’s Disease Activity Index and Pediatric Ulcerative Colitis Activity Index, respectively [11], for CD and UC patients at the time of blood sample collection for the first metabolites’ measurement, which occurred at least 3 months since the beginning of therapy. The disease was considered inactive if the disease activity index was <10 at the time of sample collection.

### 2.2. Measurement of Azathioprine Metabolites

Metabolites (TGN and MMPN) were measured in patients’ erythrocytes using the high performance liquid chromatography assay by Dervieux and Boulieu [12]. The ratio between TGN and the dose of azathioprine was calculated considering, for each individual measurement of the metabolites, the dose the patients took the day the blood sample was recorded.

### 2.3. Genotypes

Genomic DNA was extracted from peripheral blood samples using a commercial kit (Sigma, Milan, Italy), to characterize genetic polymorphisms in the candidate genes *TPMT* (rs1142345, rs1800460 and rs1800462), *GSTA1* (rs3957357), *GSTM1* (deletion), and *GSTT1* (deletion). The considered genotypes and method of analysis are described in Table 1. Genotypes for *TPMT* rs1800462 was determined by polymerase chain reaction (PCR) with allele specific oligonucleotides (ASO). Primers used were, for the wild-type allele, as forward P2W 5′-GTATGATTTTATGCAGGTTTG-3′ and as reverse P2C 5′-TAAATAGGAACCATCGGACAC-3′; primers. For the variant allele, a second tube was used with P2M 5′-GTATGATTTTATGCAGGTTTC-3′ as forward primer and the above-mentioned P2C 5′-TAAATAGGAACCATCGGACAC-3′ as reverse. PCR protocol for these primers were: initial denaturation 5 min at 94 °C, followed by 37 cycles with 30 s at 94 °C, 30 s at 57 °C, and 2 min at 72 °C, with a final extension for 10 min at 72 °C. PCR product was visualized on a 2% agarose gel. In case of a patient carrying the wild type allele, the product (254 bp) was present with the P2W and P2C primers; in case of patients carrying the variant allele, with the P2M and P2C primers for *TPMT* rs1800460 and rs1142345, PCR- restriction fragment length polymorphism (RFLP) was used. For rs1800460, primers used were: forward 5′-AGGCAGCTAGGGAAAAAGAAAGGTG-3′ and reverse 5′-CAAGCCTTATAGCCTTACACCCAGG-3′. PCR protocol for these primers was: initial denaturation 5 min at 94 °C, followed by 37 cycles with 30 s at 94 °C, 30 s at 55 °C, and 2 min at 72 °C, with a final extension for 10 min at 72 °C. The DNA amplification produces an amplicon of 694 bp, which is subsequently digested enzymatically with the enzyme Mwol (concentration of 1 U/10 μl) incubated for 90 min at 60 °C. The enzyme recognizes the wild-type site and cuts the DNA strand into two fragments of 443 bp and 251 bp, while it does not cut the variant fragment. A 2% agarose gel was prepared for visualization. For rs1142345, primers used were forward 5′-AATCCCTGCTGTCATTCTTCATAGTATTT-3′ and reverse 5′-CACATCATAATCTCCTCTCC-3′. PCR protocol was the same as *TPMT* rs1800460. PCR produces a 401 bp amplicon, which is subsequently digested enzymatically with the Accl enzyme (concentration 1 U/10 μl) and incubated for 90 min at 37 °C. The enzyme recognizes the variant site and cuts the DNA strand into two 252 pb and 149 pb fragments while the wild-type strand is not cut. A 2% agarose gel was prepared for visualization. *GSTM1* and *GSTT1* genotypes were determined by MULTIPLEX-PCR-ASO as previously described [13], in which three pairs of primers were used simultaneously: a specific pair for the T isoform, one for the M isoform and one for the β-globin gene, which acts as an internal positive control in order to verify the amplification. The three pairs of primers lead to three fragments of different sizes: 480 bp (*GSTT*), 286 bp (β-globin), and 219 bp (*GSTM*). The primers used have the following sequence: *GSTM* Forward: 5′-GAACTCCCTGAAAAGCTAAAGC-3′; *GSTM* Reverse: 5′-GTTGGGCTCAAATATACGGTGC-3′; *β-Globin* Forward: 5′-GAAGAGCCAAGGACAGGT-3′; *β-Globin* Reverse: 5′-CAACTTCATCCACGTTCACC-3′; *GSTT* Forward: 5′-TTCCTTACTGGTCCTCACATCTC-3′; *GSTT* Reverse: 5′-TCACCGGATCATGGCCAGCA-3′. PCR protocol for these primers were: initial denaturation 5 min at 94 °C, followed by 37 cycles with 30 s at 94 °C, 30 s at 57 °C, and 2 min at 72 °C, with a final extension for 7 min at 72 °C. All PCR reactions described were carried out using RedTaq polymerase (Sigma, Milan, Italy), with the addition of dNTPs 0,25 nM and with a primer concentration of 1 mM.

For *GSTA1*, pyrosequencing was employed (Table 1), since this genotyping method was already validated in the laboratory. The primers used for the pyrosequencing were: forward 5′-ATCCAGTAGGTGGCCCCTTG-3′, reverse 5′-ACCGTCCTGGCTCGACAA-3′ (biotinylated). Sequencing primer was: 5′-GCTTTTCCCTAACTTGAC-3′. PCR protocol for these primers were: initial denaturation 10 min at 95 °C, followed by 40 cycles with 30 s at 95 °C, 30 s at 66 °C, and 30 s at 72 °C and with a final extension for 10 min at 72 °C. PCR produces a 148 bp amplicon. For pyrosequencing, we used PSQ96MA (Qiagen, Hilden, Germany). PCR amplifications were performed in an Eppendorf Mastercycler gradient, with TaqGold DNA Polymerase (AB Applied Biosystems, Foster City, CA, USA).

### 2.4. Statistical Analysis

Statistical analysis was performed using the software R (version 2.15). The association between pharmacological phenotypes of interest (i.e., clinical efficacy of treatment, dose of azathioprine, TGN metabolites concentrations, MMPN metabolites concentrations, ratio TGN/dose) and the considered demographic variables, IBD type, cotreatment or genotypes in a univariate analysis, was evaluated using linear mixed effects model built using the phenotype as the dependent variable, each covariate as the fixed effect and the patients as the random effect in the model. For clinical efficacy, the first available measurement was used, while for other pharmacological variables, all available measurements were used.

Multivariate analysis was carried out to test the independence of the effects of the genotypes significant in the univariate analysis on the phenotypes considered (i.e., TGN or MMPN concentrations, dose of azathioprine, ratio TGN/dose); for this multivariate analysis generalized linear models of the Gaussian family were used considering individually each phenotype from the univariate analysis as the dependent variable and the covariates significant in the univariate analysis as the independent variables. Normality of the phenotype was tested by the Shapiro test and log10 transformation was applied if needed, in order to adjust the normality of the distribution.

## 3. Results

### 3.1. Patients Enrolled and Samples Collected

111 patients were enrolled from March 2004 to February 2015; median age was 15.05 years (IQR 12.28–16-82), 52 (46.8%) were females. Clinical and demographic characteristics of the enrolled patients are reported in Table 2.

Azathioprine metabolites were measured in 257 samples (median 2 per patient, IQR 1-3). Among these, 89 were obtained during treatment with azathioprine alone and 161 during treatment with azathioprine and other medications and in particular: 93 with an aminosalicylate, 18 with an aminosalicylate and a glucocorticoid, 15 with infliximab, 10 with an antibiotic, 4 with an aminosalicylate and an antibiotic, 4 with an antibiotic and a glucocorticoid, 3 with an aminosalicylate, an antibiotic and a glucocorticoid, 2 with an infliximab and a glucocorticoid, and 1 with an aminosalicylate and infliximab; for 7 patients, information about concomitant treatment could not be retrieved.

### 3.2. Measurement of Azathioprine Metabolites: Association with Demographic and Clinical Covariates

Results of measurements together with azathioprine dose are shown in Table 3.

**Figure 1:**
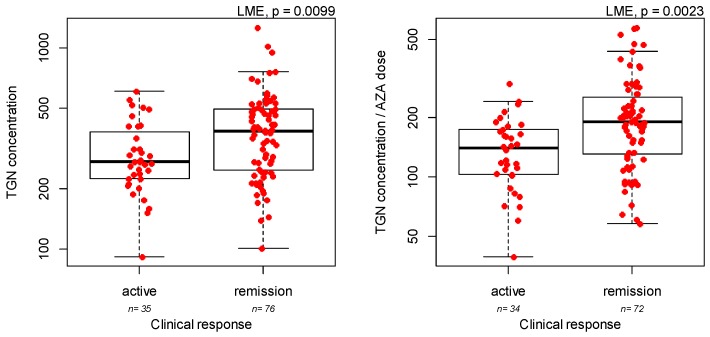
Response to azathioprine (AZA) and thioguanine nucleotides (TGN) concentration, as pmol/8 × 10^8 erythrocytes (left panel) or ratio between TGN concentration/daily azathioprine dose as pmol/8 × 10^8 erythrocytes/mg/kg/day (right panel). *p*-values are from linear mixed effect model (LME).

Concentration of TGN metabolites were associated with IBD type, with UC patients showing slightly increased concentrations (Supplementary Figure S1, LME *p* = 0.047), but not with gender or treatment length. Azathioprine dose was strongly associated with age, with younger patients taking higher doses (Supplementary Figure S2, LME *p* = 0.0001), but not with gender, IBD type or treatment length. Concentration of MMPN metabolites or the ratio between TGN concentration and azathioprine dose were not associated with IBD type, gender, or treatment length. Interestingly, the ratio between TGN concentration and azathioprine dose was strongly associated with azathioprine dose when the analysis was limited to pediatric patients (i.e., with age less than 18, Supplementary Figure S3, LME *p* = 0.0043). Clinical efficacy, defined as disease activity score below 10 at the time of first sample collection for measurement of azathioprine metabolites, was assessed in all patients. Azathioprine was more effective in UC than in CD patients (88.0% versus 52.5% responders, LME *p* = 0.0003), while gender, age, and duration of azathioprine treatment were not associated with azathioprine efficacy. A higher concentration of TGN metabolites at the first evaluation was observed in patients in remission (Figure 1, LME *p* = 0.0099), similarly a positive correlation was observed with TGN/dose ratio (Figure 1, LME *p* = 0.0023).

On the contrary, azathioprine dose and the concentration of MMPN metabolites were not associated with a clinical response (data not shown).

### 3.3. Genotyping

Results of genotyping are reported in Table 4.

**Figure 2:**
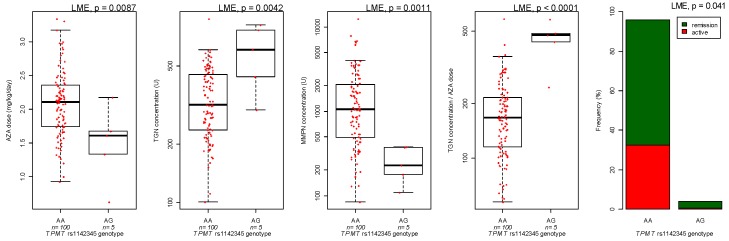
*TPMT* rs1142345 and azathioprine (AZA) dose, thioguanine (TGN) and methylmercaptopurine (MMPN) metabolites and efficacy. Concentration of azathioprine metabolites is expressed as pmol/8 × 10^8 erythrocytes (U). *p*-values are from linear mixed effect model (LME).

All polymorphisms evaluated respected Hardy-Weinberg equilibrium and their distribution was in accordance with literature data for subjects of Caucasian ethnicity. For the association between genetic variants and azathioprine pharmacokinetics, *TPMT* rs1142345 variant (4.8% of patients) was associated with increased TGN (LME *p* = 0.0042), TGN/dose ratio (LME *p* < 0.0001), decreased azathioprine dose (LME *p* = 0.0087) and MMPN (LME *p* = 0.0011; Figure 2), as well established [2]. Interestingly, all patients with variant *TPMT* were in remission at the first evaluation of thiopurine metabolites, in comparison to 65% of patients with wild-type *TPMT* (LME *p* = 0.041, Figure 2).

**Figure 3:**
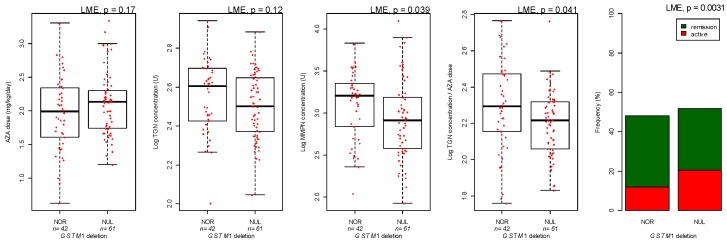
*GSTM1* deletion and azathioprine (AZA) dose, thioguanine (TGN), and methylmercaptopurine (MMPN) metabolites and efficacy. Concentration of azathioprine metabolites is expressed as pmol/8 × 10^8 erythrocytes (U). *p*-values are from linear mixed effect model (LME).

*GSTM1* deletion (58.1% of patients) was associated with a 18.5% decrease in TGN/dose ratio (LME *p* = 0.041, Figure 3) and 30% decrease in clinical efficacy (LME *p* = 0.0031; Figure 3). Additionally, MMPN was reduced in patients with deletion of *GSTM1* (LME *p* = 0.039; Figure 3).

**Figure 4:**
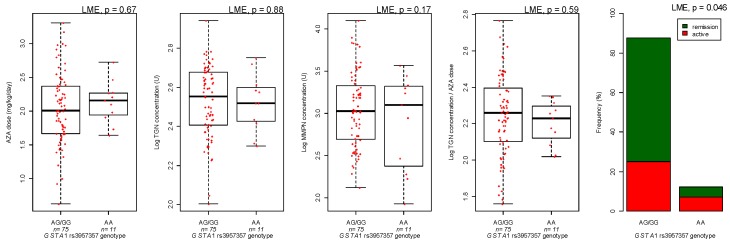
*GSTA1* rs3957357 variant and azathioprine (AZA) dose, thioguanine (TGN) and methylmercaptopurine (MMPN) metabolites and efficacy. Concentration of azathioprine metabolites is expressed as pmol/8 × 10^8 erythrocytes (U). *p*-values are from linear mixed effect model (LME).

*GSTA1* variant (12.8% of patients) showed a trend for an association with decreased clinical efficacy (LME *p* = 0.046, Figure 4); however, no significant effect on azathioprine pharmacokinetics could be detected (Figure 4).

*GSTT1* deletion was not associated with azathioprine pharmacokinetics and efficacy (data not shown). Multivariate analysis supported the results of the univariate analysis (Table 5).

## 4. Discussion

Despite the introduction of new and effective biologics in the therapy of IBD, the thiopurine drugs azathioprine and mercaptopurine continue to be frequently used for maintaining remission in these diseases. The problem with these drugs is that they are ineffective in a significant percentage of patients, and also induce side effects that can be severe [2]. The reasons for this variability are not clear; however, a number of studies have suggested that variations in enzymes involved in their metabolism can be involved.

For azathioprine, this agent is the prodrug of mercaptopurine, and has to be converted to produce its pharmacological activity. This conversion can occur spontaneously, but is also catalyzed by the enzymes GST, in particular the A and M isoforms [16]. In rat liver homogenates, Kaplowitz et al. have demonstrated that, while at high pH (pH = 8.0) the nonenzymatic and enzymatic reactions occur at similar levels, at pH closer to physiological values, the enzymatic reaction prevails [17]. In addition, in homogenates of human livers obtained from transplant donors, treatment with furosemide, an inhibitor of soluble GSTs [18], inhibited the conversion of azathioprine to mercaptopurine [19]. Additional evidence of a role of GSTs in azathioprine metabolism has been obtained in animal models; indeed, pretreatment of rats with the GST inhibitor probenecid increased the proportion of azathioprine in rat liver and reduced GSH consumption. Similarly, less hepatic GSH depletion was observed after azathioprine treatment in Gunn rats, a model of hyperbilirubinemic rat [17]. Of interest, bilirubin is also a GST inhibitor, with some studies indicating a stronger inhibitory effect of bile acids on GSTM1 in comparison to other isoforms [20].

We previously showed that the frequency of *GSTM1* deletion was significantly lower in patients that developed an adverse event in comparison to patients that tolerated azathioprine treatment, in agreement with a model in which patients with *GSTM1* deletion are less sensitive to the effects of azathioprine, putatively because of the contribution of this enzyme on the conversion of azathioprine to mercaptopurine [13]. Moreover, in a recent previous study [21], we evaluated the effects of GST polymorphisms on azathioprine biotransformation in a cohort of young patients with IBD, tolerant to azathioprine therapy and taking the drug for more than 3 months. Patients with the deletion of *GSTM1* tolerated a dose of azathioprine significantly higher in comparison to patients with normal *GSTM1*. Moreover, the amount of active TGNs generated in patients with the deletion of *GSTM1* was significantly decreased in comparison to patients with a normal genotype. Multivariate analysis confirmed that this effect was independent from that of other genes with a significant effect, such as *TPMT*, the main gene known to influence mercaptopurine metabolism [22].

The present study is the first report of an association between azathioprine efficacy and *GSTM1* and *GSTA1* variants in young patients with IBD. Moreover, we confirmed the reduced TGN/azathioprine dose ratio in patients with *GSTM1* deletion we previously reported, which may be associated with the described lower efficacy of azathioprine in patients with this genotype. This could support the need for genotype adjusted tailored therapy, possibly testing the efficacy of strategies leading to higher TGN concentration in patients with *GSTM1* deletion, such as increased azathioprine dose or co-treatment with an aminosalycilate [23], even if prospective studies are needed to further support these strategies.

Therefore, all these studies support a role of *GSTM1* on azathioprine efficacy, mediated by an increased conversion of azathioprine to mercaptopurine. The reaction catalyzed by GSTM1 likely occurs after oral administration mainly in the intestine and the liver, modulating the amount of mercaptopurine and TGNs that are released in the main circulation [24].

Azathioprine dose is strongly associated with patients’ age in the present study, an observation consistent with our previous results in children with IBD, showing that these patients require higher doses of azathioprine to achieve similar therapeutic efficacy and TGN concentration [25]. *TPMT* activity indeed is significantly higher in children than in adults [26]; interestingly, in pediatric patients (age less than 18 years), we could observe a lower ratio of TGN/azathioprine dose, as in our previous report. However, when the analysis was extended to young adults (age less than 30 years), the correlation between age and the TGN/dose ratio was lost. This may be related to environmental factors, including epigenetic determinants, even if more studies are needed to elucidate the mechanisms underlying these observations [27].

Considering studies by other groups, our results are in agreement with a recently published paper describing a lower efficacy of azathioprine in patients with *GSTM1* deletion, even if the results were not fully significant [28]. Moreover, in our study, we observed reduced concentration of MMPN nucleotides during azathioprine treatment in patients with *GSTM1* deletion: this result is consistent with a recent study by Broekman et al. [29]; this study also supports a lack of effect of *GSTA1* variants on azathioprine TGN and MMPN concentrations. The clinical implications of these observations need to be further explored. Additionally, age may affect the association of GSTs variants with thiopurine effects; indeed, studies in adult patients could not identify a consistent effect of *GSTM1* variants on thiopurines induced adverse effects [30], and therefore, other studies in the pediatric population are needed.

A recent study investigated the association among *GST* polymorphism, enzyme activity and azathioprine-related adverse drug reactions in Chinese Han patients with IBD, finding that the patients who became neutropenic had a significantly higher GSTs activity when compared with patients who did not develop toxicity [31]. The authors found, in the univariate analysis, that *GSTM1* wildtype genotype had a relationship with leukopenia and flue like symptoms, while *GSTP1* variant was strongly associated with leukopenia. Following adjustment for other potential risk factors, it was shown that *GSTP1* variants only were associated with increased risks of leukopenia. In our current study, we did not consider the effect of *GSTP1* polymorphisms on azathioprine effects and metabolism, since in our previous studies no significant association with adverse effect [13] or biotransformation [21] could be detected. The lack of association may be due to the tissue distribution of *GSTP1* and *GSTT1*, which are not highly expressed in the liver, but even to the lack of specific activity of these enzymes toward the catalysis of the reaction of azathioprine with glutathione [9,16]. Since the GSTP isoform does not catalyze the biotransformation of azathioprine to mercaptopurine [16], other mechanisms could be involved in the association observed by Liu and collaborators, such as induction of oxidative stress or modulation of apoptosis [24]. The study by Liu et al. reporting an effect of *GSTP1* variants on azathioprine induced adverse events, with a milder effect of *GSTM1*, seems to underline that the effects of GSTs on azathioprine pharmacogenetics may be influenced by ethnicity. Indeed, it is already known that variants frequent in Asian patients but uncommon in other ethnic groups, are associated with increased sensitivity to thiopurines, such as *MRP4* and *NUDT15* [32,33].

One limitation of our study is its retrospective design and the consequent difficulty of properly assessing phenomena such as drug interaction, which should rely on data collection from patients’ charts. Drug interactions between azathioprine and other agents employed in IBD have been described; in particular, a significant decrease in TGN levels after discontinuation of aminosalicylates has been previously reported [23]. Moreover, for *GSTA1*, a marginal effect on thiopurine efficacy was observed but this was not supported by an effect on thiopurine pharmacokinetics: this may be related to the limited number of patients homozygous for the *GSTA1* variant enrolled. Another limitation is the fact that the current assay for thiopurine metabolites quantifies two main species (thioguanine nucleotides and methyl-mercaptopurine nucleotides), without distinguishing between monophosphate, diphosphate, and triphosphate nucleotides. Innovative mass spectrometry based assays are now available to quantify thiopurine metabolites [34], allowing quantification of phosphorylation of thionucleotides [35] and they could be applied to evaluate differences in thiopurine biotransformation in patients with various GST genotypes. Evaluation of the combined effects of genotypes in this study is limited. Indeed, multivariate analysis indicates independency in the effects of the candidate genotypes considered on the pharmacological variables in the present cohort. A larger cohort is needed to detect significant effects by combined genotypes. For demographic covariates, in particular gender, no significant effect was identified in the univariate analysis; to further evaluate interactive effect of gender and the considered genotypes, a larger cohort is needed.

In conclusion, GSTs variants were associated with azathioprine efficacy and pharmacokinetics; more studies, both clinical and molecular are still needed to apply this evidence to improve outcomes of therapy with azathioprine in young patients with IBD.

## Figures and Tables

**Table 1:** Assay used for genotyping of the considered variants.

Gene	Polymorphism		
	rs Number	Primary Locus Alleles [14]	Genotyping Method
*TPMT*	rs1800462	C > G missense	PCR-ASO [13]
	rs1800460	C > T missense	PCR-RFLP [13]
	rs1142345	T > C missense	PCR-RFLP [13]
*GSTM1*	No rs number	Deletion	MULTIPLEX-PCR-ASO [13]
*GSTT1*	No rs number	Deletion	MULTIPLEX-PCR-ASO [13]
*GSTA1*	rs3957357	A > G (5′-UTR)	Pyrosequencing [15]

ASO: allele specific oligonucleotides; *GST*: glutathione-S-transferase, *TPMT*: thiopurine-S-methyl transferase; RFLP: restriction fragment length polymorphism. PCR: polymerase chain reaction.

**Table 2:** Demographic and clinical characteristics of the patients enrolled in the study.

		All Patients (n = 111)
Age (Years) at Time of Sample Collection		15.1, 12.3–16.8
Gender	Female (%)	52 (46.8%)
	Male (%)	59 (53.2%)
Type of IBD	Crohn’s disease (%)	61 (55.0%)
	Ulcerative colitis (%)	50 (45.0%)
Length (days) of treatment with azathioprine		533, 245–917

For continuous variables, median, 1st–3rd quartiles values are reported. To report age and length of treatment median and interquartile range are provided; for patients with more than one measurement of azathioprine metabolites, median age and length of treatment were used.

**Table 3:** Summary of azathioprine’s dose and metabolites’ concentrations.

	TGN (pmol/8 × 10^8 Erythrocytes)	MMPN (pmol/8 × 10^8 Erythrocytes)	Dose (mg/kg)	TGN/Dose ((pmol/8 × 10^8 Erythrocytes)/(mg/kg))
Mean	361.6	1698.1	2.0	192.8
Median	345.0	1044.0	2.1	179.4
Interquartile range	240.1–465.1	431.2–2079.7	1.7–2.3	120.1–227.9

MMPN indicated methylated nucleotides, TGN indicates thioguanine nucleotides.

**Table 4:** Genotype distribution in the 111 patients enrolled in the study.

**Gene**	**Polymorphism**	**Genotyping Result**				
	**rs Number**	**Wild-Type**	**Hetero-zygous**	**Homozygous Variant**	**Not Available**	***p*-value Hardy Weinberg**
*TPMT*	rs1800462	105 (100%)	0	0	6	NA
*TPMT*	rs1800460	101 (97.1%)	3 (2.9%)	0	7	0.88
*TPMT*	rs1142345	100 (95.2%)	5 (4.8%)	0	6	0.81
*GSTA1*	rs3957357	38 (44.2%)	37 (43.0%)	11 (12.8%)	25	0.77
**Gene**	**Polymorphism**	**Genotyping Result**				
		**Not Deleted**		**Deleted**	**Not Available**	
*GSTM1*	Deletion	42 (41.9%)		61 (58.1%)	8	
*GSTT1*	Deletion	78 (75.7%)		25 (24.3%)	8	

GST indicates glutathione-S-transferase, *TPMT* indicates thiopurine-S-methyl transferase.

**Table 5:** Multivariate analysis considering for each pharmacological dependent variable covariate significant in the univariate analysis.

Azathioprine Related Pharmacological Phenotype (Dependent Variable)	Independent Variable in Multivariate Generalized Linear Model	Comparison	Effect	*p*-Value
Efficacy of azathioprine at the first metabolite measurement	IBD type	UC versus CD	1.96	0.0019
	*GSTM1* genotype	Deletion versus Normal	−1.49	0.019
	*GSTA1* genotype	AA versus GG/GA	−1.30	0.095
	*TPMT* genotype	AG versus GG	24.7	0.43
TGN metabolites concentrations	IBD type	UC versus CD	0.061	0.074
	*TPMT* genotype	AG versus GG	0.23	0.0049
MMPN metabolites concentration	*GSTM1* genotype	Deletion versus Normal	−0.21	0.014
	*TPMT* genotype	Heterozygous versus wild-type	−0.72	0.0004
Azathioprine dose	Age	Each year	−0.035	<0.0001
	*TPMT* genotype	Heterozygous versus wild-type	−0.58	0.0056
Ratio TGN/dose	*TPMT* genotype	Heterozygous versus wild-type	0.41	0.0001
	*GSTM1* genotype	Deletion versus Normal	−0.072	0.055

*GST*: glutathione-S-transferase, IBD: inflammatory bowel disease, UC: ulcerative colitis, CD: Crohn’s disease, MMPN: methylated nucleotides, TGN: thioguanine nucleotides, *TPMT*: thiopurine-S-methyl transferase. The effect size represents the increase (positive value) or decrease (negative value) in the value of the dependent variable for each independent variable listed. *p*-values are from linear mixed-effect models.

## Supplementary Materials

The following are available online at https://www.mdpi.com/2073-4425/10/4/277/s1, Figure S1: inflammatory bowel disease (IBD type) and azathioprine thioguanine nucleotide (TGN) metabolites, Figure S2: Patient’s age and azathioprine daily dose, Figure S3: Patient’s age and ratio between concentration of azathioprine thioguanine-nucleotide metabolites and azathioprine daily dose.
