# Supplementary material for: Azathioprine Biotransformation in Young Patients with Inflammatory Bowel Disease: Contribution of Glutathione-S Transferase M1 and A1 Variants
Source: Genes (Basel). 2019 Apr 4;10(4):277. doi: 10.3390/genes10040277 (PMC6523194; doi:10.3390/genes10040277)
Supplement: Supplementary file 1 [file genes-10-00277-s001.pdf]

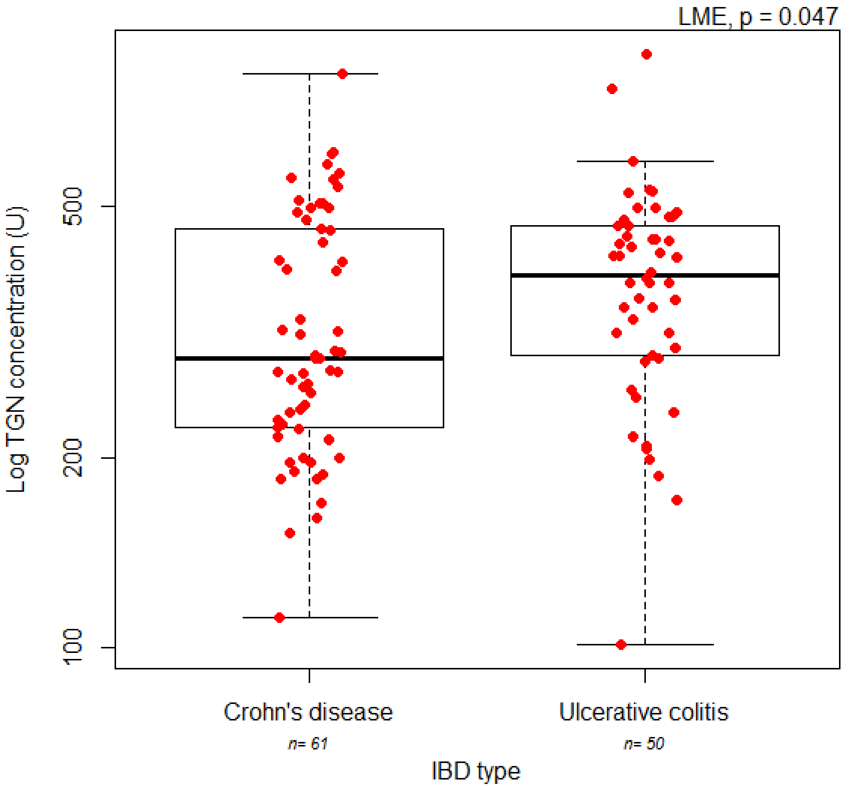

Supplementary Figure 1: inflammatory bowel disease (IBD type) and azathioprine thioguanine nucleotide (TGN) metabolites. Concentration of azathioprine metabolites is expressed as pmol / 8 x 10^8 erythrocytes (U). P-values are from linear mixed effect model (LME).

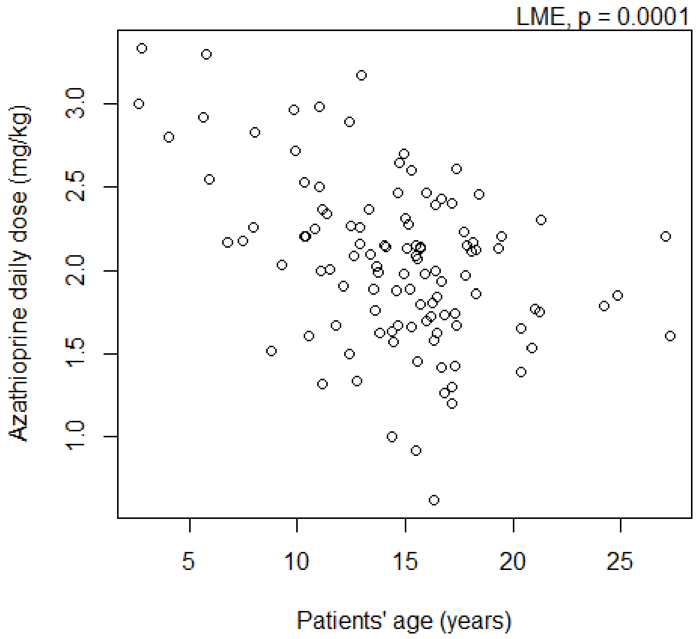

Supplementary Figure 2: Patient’s age and azathioprine daily dose (mg/kg). P-values are from linear mixed effect model (LME).

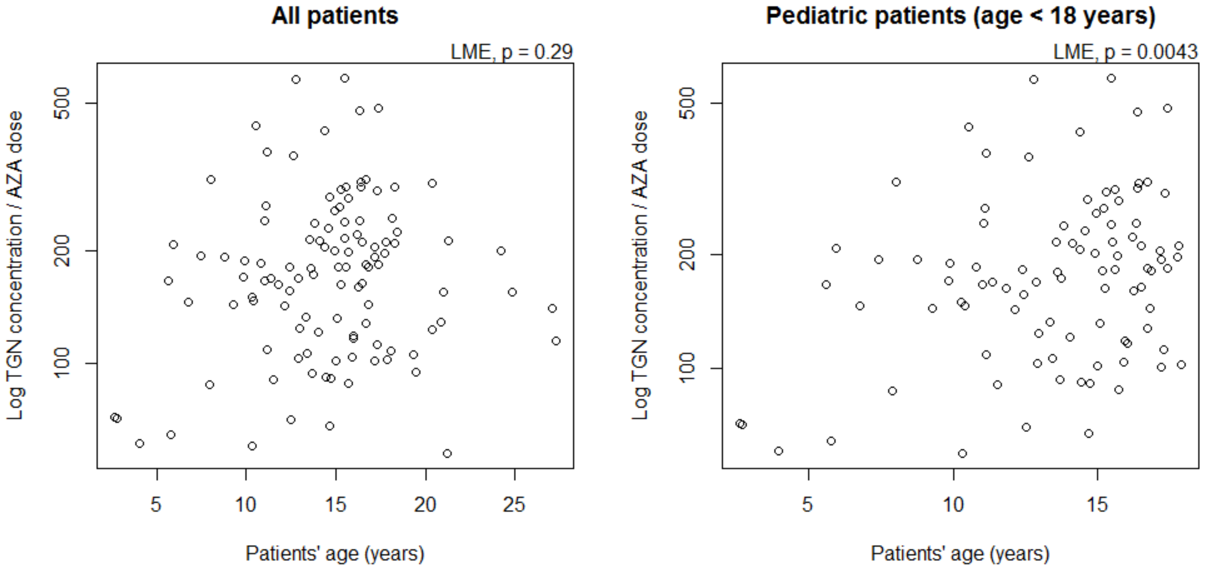

Supplementary Figure 3: Patient's age and ratio between concentration of azathioprine (AZA) thioguanine-nucleotide metabolites (TGN) and AZA daily dose. P-values are from linear mixed effect model (LME). Left panel shows data from all patients, while right panel displays data from pediatric patients (age less than 18 years).
